# *In Silico* Determination and Validation of Baumannii Acinetobactin Utilization A Structure and Ligand Binding Site

**DOI:** 10.1155/2013/172784

**Published:** 2013-09-05

**Authors:** Fatemeh Sefid, Iraj Rasooli, Abolfazl Jahangiri

**Affiliations:** ^1^Department of Biology, Shahed University, Tehran-Qom Express Way, Tehran 3319118651, Iran; ^2^Applied Microbiology Research Center, Baqiyatallah University of Medical Sciences, Tehran 1435944711, Iran

## Abstract

*Acinetobacter baumannii* is a deadly nosocomial pathogen. Iron is an essential element for the pathogen. Under iron-restricted conditions, the bacterium expresses iron-regulated outer membrane proteins (IROMPs). Baumannii acinetobactin utilization (BauA) is the most important member of IROMPs in *A. baumannii*. Determination of its tertiary structure could help deduction of its functions and its interactions with ligands. The present study unveils BauA 3D structure via *in silico* approaches. Apart from *ab initio*, other rational methods such as homology modeling and threading were invoked to achieve the purpose. For homology modeling, BLAST was run on the sequence in order to find the best template. The template was then served to model the 3D structure. All the models built were evaluated qualitatively. The best model predicted by LOMETS was selected for analyses. Refinement of 3D structure as well as determination of its clefts and ligand binding sites was carried out on the structure. In contrast to the typical trimeric arrangement found in porins, BauA is monomeric. The barrel is formed by 22 antiparallel transmembrane **β**-strands. There are short periplasmic turns and longer surface-located loops. An N-terminal domain referred to either as the cork, the plug, or the hatch domain occludes the **β**-barrel.

## 1. Introduction

*Acinetobacter baumannii* is a causative pathogen for severe infections. Treatment of infections caused by this pathogen is a significant problem in human medicine [1]. Iron is an essential element for most pathogens including *A. baumannii*. Low solubility of the essential element under aerobic conditions or at physiological pH imposes limiting condition to the bacterium [1]. Because of its insufficient free concentration in biological fluids, the bacterium employs strategies to obtain iron in complex forms. Iron-regulated outer membrane proteins (IROMPs) are expressed under iron-restricted conditions in the bacterium [2]. These proteins are also expressed in other bacteria like *Pseudomonas aeruginosa*, *Escherichia coli*, and *Neisseria gonorrhoeae*. These proteins are members of outer membrane proteins (OMPs) and are structurally and functionally different from open porins [3]. Smaller molecules could pass through these porins in the outer membrane (OM). However, molecules above 600 Da must use outer OMP to pass the layer [4]. Siderophores are components that act as an iron chelator. The molecules, scavenging iron from proteins of the host, are synthesized and secreted by most bacteria under iron-limiting conditions. Molecular weight of iron-siderophore complexes is more than 600 Da.; therefore, these require IROMPs for entry into the cell [5]. Baumannii acinetobactin utilization; (BauA) is the most important member of IROMPs in *A. baumannii* playing a pivotal role in uptake of acinetobactin, the siderophore of *A. baumannii*, in complex with iron under iron-limiting conditions [2]. Lack of the acinetobactin outer membrane receptor is followed by siderophore utilization disruption resulting in iron uptake deficiency. Disruption of the BauA function is followed by growth inhibition under iron-restricted conditions [6]. In addition to bactericidal and opsonizing activity, monoclonal antibodies generated against IROMPs can block the iron uptake system *in vitro *[7]. A greater understanding of the nature of BauA and also its role in serious infections of *A. baumannii* will help develop new and more effective treatment for *Acinetobacter *infections. The knowledge of tertiary structure of proteins could help deduction of their functions and also their interactions with other compounds such as ligands [8]. Moreover, rational modification and engineering of proteins depend on understanding their 3D structures [9]. 3D protein structures could be employed in drug and vaccine designs [10] and conformational epitope predictions [11]. The huge number of known protein sequences versus the inconsiderable number of structural annotations highlight necessity of identification of tertiary protein structures [12]. Experimental determination of protein structures remains an important challenge due to its high failure rates. Since experimental determination of 3D protein structures is expensive and time consuming, other approaches are ought to be considered [13]. For outer membrane proteins, purification and crystallization are further obstacles in addition to common experimental determination of 3D protein structures. Nowadays, bioinformatic tools are of interesting advantages for biologists [14, 15]. Prediction of 3D protein structure is one of the wide applications of these tools [16, 17]. Several methods and algorithms are available for protein structure predictions, homology modeling being one of them. Homology modeling is an *in silico* method for prediction of 3D protein structures based on known homologous protein structures as a template. Crystallographic structures of several BauA homologous proteins such as FhuA, FpvA, FptA, and FepA in other pathogens have been documented [18]. However 3D structure of BauA is yet to be determined. The present study undertakes this task.

## 2. Methods

### 2.1. Sequence Availability

The BauA protein sequence with accession no. AAT52186.1 obtained from NCBI [19] at http://www.ncbi.nlm.nih.gov/protein was saved in FASTA format for further analyses.

### 2.2. Homology Search

The BauA sequence served as a query for BLAST [20, 21] at http://blast.ncbi.nlm.nih.gov/Blast.cgi against nonredundant protein database. Probable putative conserved domains of BauA were also searched for, at the above address.

### 2.3. Template Search

The protein sequence of BauA was used as an input data for the PSI-BLAST against protein data bank (PDB) [22] at http://blast.ncbi.nlm.nih.gov/Blast.cgi to identify its homologous structures. 

### 2.4. Sequence Alignments

Ten protein sequences of *A. baumannii* with *E* value = 0 and identity > 60% obtained from template search were aligned for precise analysis of homology. To evaluate validity of the structurally related sequences, the amino acid sequence of BauA was aligned against template sequences from previous template search. PRALINE [23] at http://www.ibi.vu.nl/programs/pralinewww/ was used to align the BauA and the selected template, that is, FptA sequences. The BLOSUM substitution matrix (BLOSUM62) was selected with a gap penalty of 12 and a gap extension penalty of 1.

### 2.5. Topology Prediction

Prediction of the hydrophobic transmembrane regions in a protein sequence forming probable *β*-barrel could help the determination of the 3D protein structure. Full-length as well as the truncated protein (from amino acid 212 for FptA template and amino acid 192 for the BauA query) served as inputs in topology predictions. PRED-TMBB [24] (http://biophysics.biol.uoa.gr/PRED-TMBB/) is a sever that predicts transmembrane *β*-strands in protein sequences of Gram-negative bacteria. The web server could find the topology of the loops in addition to localizing the transmembrane strands [24].

### 2.6. Secondary Structure Prediction

Self-optimized prediction method (SOPM) has been described to improve the success rate in the prediction of the secondary structure of proteins. The secondary structure of the protein was predicted by SOPMA [25] at http://npsa-pbil.ibcp.fr/cgi-bin/npsa_automat.pl?page=npsa_sopma.html. Parameters of this server were set as follows: numbers of conformational states 4 (helix, sheet, turn, and coil), similarity threshold 8, and window width 17.

### 2.7. Homology Modeling

In the process of BauA structure modeling, default restraint settings were applied, and a rigorous relaxation protocol involved 2000 simulated annealing relaxation cycles (4.4 ps stepwise warming from 0–1000 K, followed by 19.2 ps stepwise cooling back down to 300 K, all done through Charmm force field and charges). The loop regions geometry was corrected using MODELER/Refine Loop command.

 (PS)^2^ [26] at http://ps2.life.nctu.edu.tw/ is an automated homology modeling server. The method uses an effective consensus strategy by combining PSI-BLAST, IMPALA, and T-Coffee in both template selection and target-template alignment. 

ESyPred3D [27] at http://www.fundp.ac.be/sciences/biologie/urbm/bioinfo/esypred/ is a new automated homology modeling program. The method using neural networks is benefited by the increased alignment performances of a new alignment strategy.

The automated system, 3D-JIGSAW [28] at http://bmm.cancerresearchuk.org/~3djigsaw/, is to build three-dimensional models for proteins based on homologues of known structure. The program searches for homologous templates in our sequence databases (PFAM + PDB + nr) and splits the query sequence into domains. If good templates are found, the best covered domain is then modeled using a maximum of 2. Geno3D [29] (http://geno3d-pbil.ibcp.fr) is an automatic web server for protein molecular modelling.

### 2.8. Other Modeling Methods

I-TASSER [30, 31] server at http://zhanglab.ccmb.med.umich.edu/I-TASSER/ predicts protein structures and functions. 3D models are built based on multiple-threading alignments as well as *ab initio*. LOOPP (Learning, Observing, and Outputting Protein Patterns) [32] server at http://cbsuapps.tc.cornell.edu/loopp.aspx is a fold recognition program based on the collection of numerous signals, merging them into a single score and generating atomic coordinates based on an alignment into a homologue template structure. The signals include straightforward sequence alignment, sequence profile, threading, secondary structure, and exposed surface. Phyre2 [33] at http://www.sbg.bio.ic.ac.uk/phyre2/html/page.cgi?id=index uses the alignment of the hidden Markov models via HHsearch to significantly improve accuracy of alignment and detection rate. Phyre2 also incorporates a new *ab initio* folding simulation called Poing to model regions of proteins with no detectable homology to known structures. LOMETS [34] server at http://zhanglab.ccmb.med.umich.edu/LOMETS/ (Local Meta-Threading-Server) is an online web service for protein structure prediction. It generates 3D models by collecting high-scoring target-to-template alignments from 10 locally installed threading programs (FUGUE, HHsearch, MUSTER, PPA, PROSPECT2, SAM-T02, SPARKS^X^, SP3, FFAS, and PRC).

### 2.9. Models Evaluations

All 3D models of BauA structure were qualitatively estimated by 4 various independent servers (PROSESS,VADAR, PROSA, and Qmean). PROSESS [35] server at http://www.prosess.ca (Protein Structure Evaluation Suite & Server) is a web server designed to evaluate and validate protein structures solved by either X-ray crystallography or NMR spectroscopy. Energy-minimized models assigned to pH of extracellular fluid, that is, 7.4, resulted in a +1 charge for arginines and lysines and −1 charge for the aspartates and glutamates. The stereo chemical quality of structures models was evaluated using online version of VADAR 1.8 (Volume Area Dihedral Angle Reporter) [36] at http://redpoll.pharmacy.ualberta.ca/vadar. The server could also depict the Ramachandran plot. ProSA [37] specifically addresses the needs encountered in the validation of protein structures obtained from X-ray analysis, NMR spectroscopy, and theoretical calculations. ProSA-web is accessible at https://prosa.services.came.sbg.ac.at.

QMEAN [38] at http://swissmodel.expasy.org/qmean/cgi/index.cgi?page=help is a composite scoring function which is able to derive both global (i.e., for the entire structure) and local (i.e., per residue) error estimates on the basis of one single model.

In addition to employing the above-mentioned servers for quality evaluation of the predicted 3D structures, the Ramachandran plots were also depicted for each model by Rampage at http://mordred.bioc.cam.ac.uk/~rapper/rampage.php.

### 2.10. Model Refinement

ModRefiner [39] at http://zhanglab.ccmb.med.umich.edu/ModRefiner/ is an algorithm for atomic-level, high-resolution protein structure refinement, which can start from either C-alpha trace, main-chain model or full-atomic model. The server improves the physical realism and structural accuracy of protein models by a two-step atomic-level energy minimization and produces a PDB. The PDB produced thereby is then analyzed by the Ramachandran plot. BauA predicted structure was refined by this server.

### 2.11. Structure Alignment

The best predicted 3D structure of the protein was structurally aligned with the most appropriate template (PDB code: 3qlB) by Dali [40, 41] at http://ekhidna.biocenter.helsinki.fi/dali_server/. This server calculates multiple structural alignment: A multiple 3D alignment compares several structures belonging to the same superfamily, which provides important biological insight such as conserved sites or conserved structural features. Structure alignment were carried out between BauA and FptA (PDB code: 3qlB), FepA (PDB code: 1FEP), FhuA (PDB code: 1by5), FecA (PDB code: 1pnz), FpvA (PDB code: 1xkh), and BtuB (PDB code: 1NQE). The outputs of a structural alignment are superposition of the atomic coordinate sets and a minimal root mean square deviation (RMSD) between the structures. The RMSD of two aligned structures indicates their divergence from one another. The differences in relative orientation of the domains between two structures to be aligned are artificially inflated the RMSD.

### 2.12. Data Validation

All bioinformatic procedures performed for the query (BauA) were also carried out on the template (FptA) protein. This task was accomplished to compare the results obtained from the servers and to validate the results on the query protein.

### 2.13. Ligand Binding Site Predictions

Cofactor [42] at http://zhanglab.ccmb.med.umich.edu/COFACTOR/ is a structure-based method for biological function annotation of protein molecules. Important amino acid involved in ligand binding site is predicted by this server.

### 2.14. Cleft Analyses

Profunc [43] at http://www.ebi.ac.uk/thornton-srv/databases/profunc/ was used to predict clefts and grooves in the protein surface. This server also predicts depth of clefts and amino acids that are located at the clefts.

## 3. Results

### 3.1. BLAST Results

BLAST search revealed numerous hits to BauA sequence. Several hits were of bacteria other than *Acinetobacter. *Putative conserved domains were detected within the sequence. Most of the sequences belonged to TonB dependent/ligand-gated channels, ligand-gated-channel protein family, and outer membrane-channels superfamily.

### 3.2. Template Selection

Table 1 shows the first 10 hits with the highest scores of the BLAST on the query sequence against protein data bank (PDB). The first hit (accession: 3QLB-A, chain A, enantiopyochelin outer membrane tonb-dependent transporter from *Pseudomonas fluorescens* bound to the ferri-enantiopyochelin [44], max. score: 92.8, Query coverage: 94%, max. ident: 24%) possessing the highest score was selected as a template.

### 3.3. Alignments

**Figure 1:**
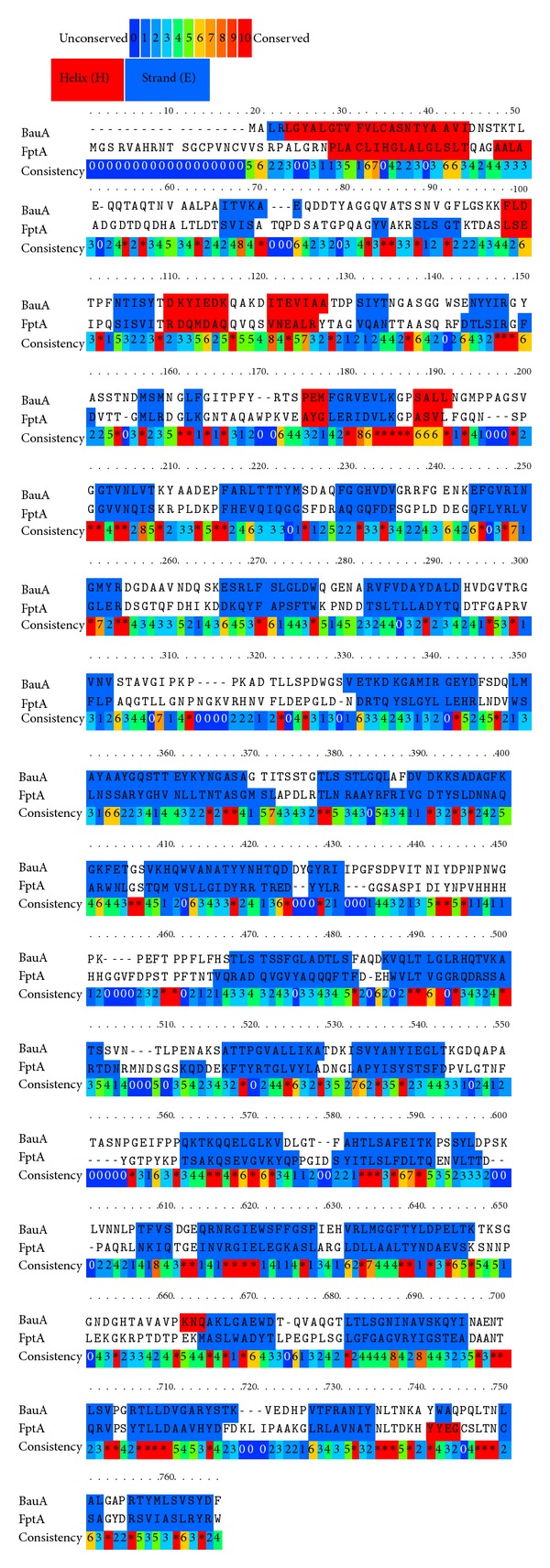
Illustration of homology between the BauA sequence and the selected template FptA (accession: 3QLB_A and GI: 359545762) from *Pseudomonas fluorescens*. The superposition was made with the praline program and adjusted manually. Conserved residues are highlighted from blue to red colors in the nether line.

No significant difference was noted between the BauA sequence in *Acinetobacter* species. A schematic illustration of homology between the BauA sequence and the selected template from *Pseudomonas fluorescens* (Figure 1) shows 20% identity.

### 3.4. *In Silico* Topology Modeling

**Figure 2:**
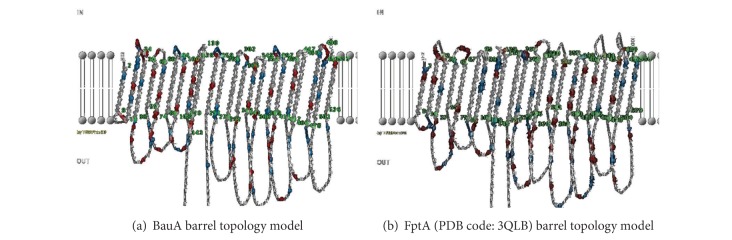
A 2D topology model of BauA and FptA. Both whole proteins include 2 domains comprising a cork domain at N terminal of the protein and a trans membrane barrel at the C terminal. These pictures show only the barrel topology composed of 22 trans membrane beta sheet, 10 short periplasmic turns, and 11 large extracellular loops. In both predicted topology, the loop number 5 is the largest and loop number 1 is the shortest.

A 2D topology model of BauA was built based on predicted inside, transmembrane, and outside regions of the protein (Figure 2). This protein is composed of 22 transmembrane antiparallel *β*-strands. The model suggests that the protein possesses *β*-barrel structure in native form. Strands forming *β*-barrel are linked together through loops at the outside or turns inside. About 30 residues at the N-terminus are predicted inside from topology point of view.  Table 2 shows the statistics of conserved residue in cork and barrel domains in BauA.

### 3.5. Secondary Structure

Coil, helix, and strands are components constituting secondary structure of the protein. The secondary structure could be used to validate the tertiary structures. Attributions of the secondary structure components in the protein are alpha helix (18.21%), extended strand (21.79%), beta turn (4%) and random coil (56%). 

### 3.6. 3D Modeling

Each server and software recruited for homology modeling independently introduced one model. All the models were selected for further analyses. I-TASSER built 4 models which ranked based on their C-scores. Among the offered models, the best with the highest C-score was selected for validation analyses. Five models were predicted by LOOPP server. All the models were obtained for the next steps. Phyre2 predicted one three-dimensional model and LOMETS meta server predicted 10 models for the protein. The models were also taken for further analyses.

### 3.7. Evaluation of Models

The 3D models estimated qualitatively by four various independent servers revealed a consensus on a single model (Table 3). PROSA *z*-scores for the most similar structure, was −4.62 and the *z*-scores of the best model calculated was −4.02. *z*-scores of all other models were not within the favorable range. 

The most valuable Qmean score was 0.440 for the best model as compared with FptA Qmean score of 0.508 selected for comparison. In the Ramachandran plot of models the percent residues were located in favored, allowed, and outlier regions. Amongst all the predicted models, the selected model was outstanding. With the maximum percent of favored residue (94.5%) and the minimum percent of outlier residue (1.1%) this model was superior to that of FptA. PROSESS server estimated covalent bond, noncovalent packing, and torsion angle quality. These indices in the best model were calculated as 7.5, 5.5, and 7.5, respectively, as against those of FptA as 7.5, 6.5, and 4.5. A model in consensus of all the servers was selected as the best 3D model. This model was built by LOMETS.

In predicted model the excluded volume calculated by VADAR was 1.0 (sd = 0.1). 

### 3.8. Model Refinement

**Figure 3:**
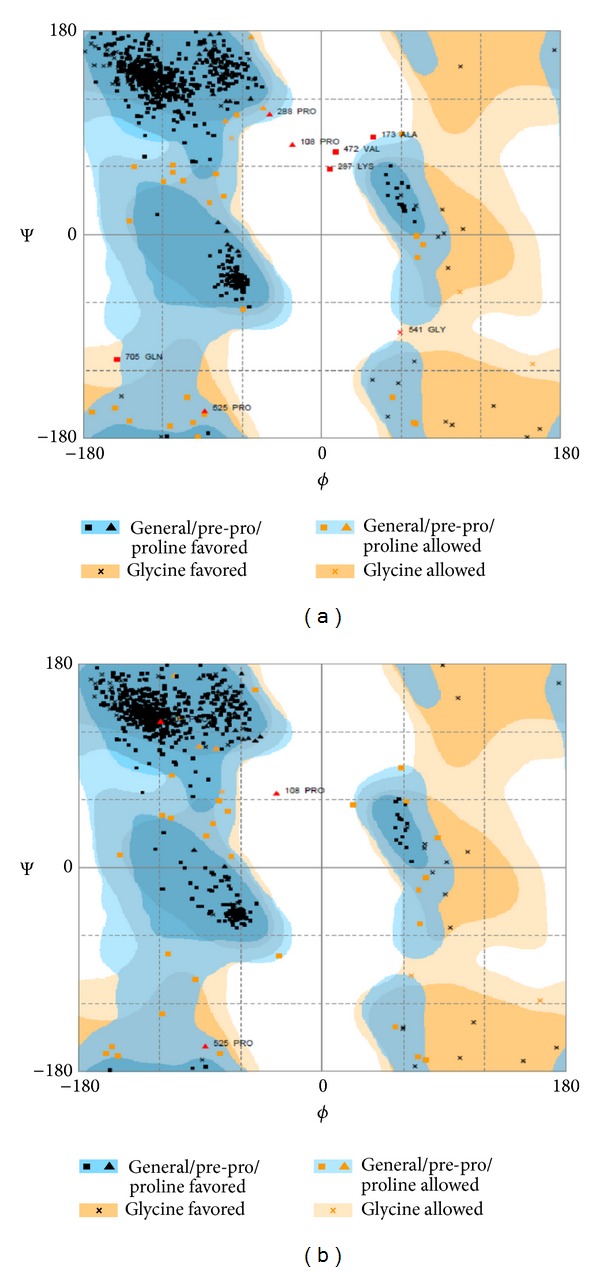
Ramachandran plot of initial and the final BauA models after refinement. (a) Number of residues in favored region: 683 (94.5%). Number of residues in allowed region: 32 (4.4%). Number of residues in outlier region: 8 (1.1%). (b) Number of residues in favored region: 685 (94.7%). Number of residues in allowed region: 35 (4.8%). Number of residues in outlier region: 3 (0.4%).

The Ramachandran plots of the initial and final models were depicted and compared after refinement (Figure 3). In initial model the percent residues in the favored region were 94.5% while 94.7% in the final model. Percent residues in allowed region for initial and final models are 4.4% and 4.8%, respectively. Eight (1.1%) residues in outlier region in the initial model decrease to 3 (0.4%) in the final model. The Ramachandran plot (Figure 3) indicates that some amino acids in the best predicted structure are located at outlier region. 

### 3.9. Structures Alignments

**Figure 4:**
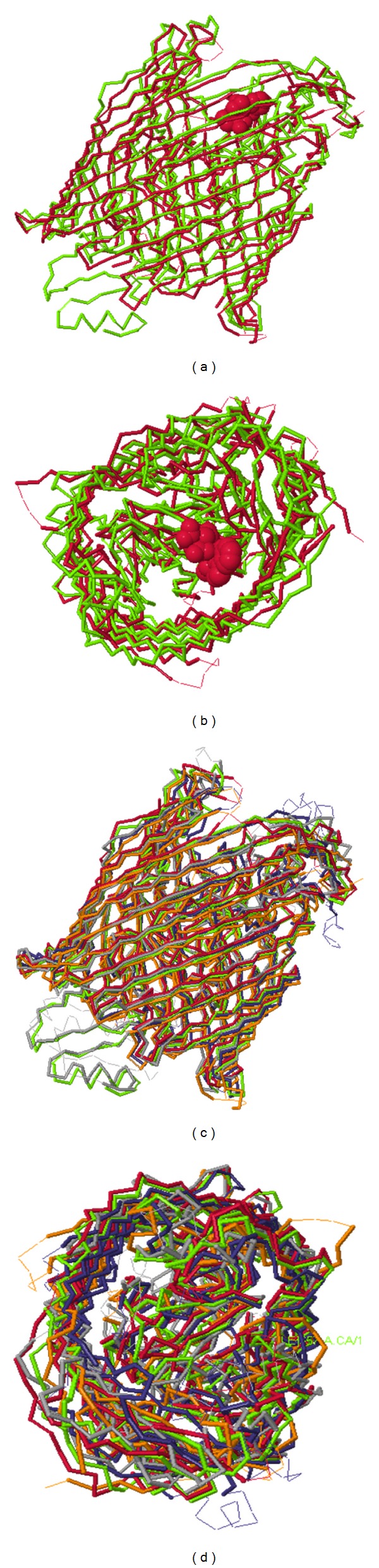
Dali 3D structure alignment between query (BauA) and template (FptA). (a) and (b) Structure alignment between BauA (green) and FptA (red) from lateral and top views, respectively. Ligand appeares in space filling model in red color. Ligand binding site is predicted on the cork domain. (c) and (d) Structure alignment between BauA (green), FptA (red), FepA (orange), FhuA (grey), FecA (blue), FpvA (violet), and BtuB (pink) from lateral and top views, respectively.

Based on Dali Structures alignments no significant discrepancy was seen between the template and query 2D and 3D structures. Majority of tertiary structure of the template and query was matched in tertiary structure alignment (Figure 4).

Expansion of structure alignment is based on distance criteria exclusively. Neither the statistical significance of the alignment nor the size of gaps was regarded in this analysis. The longest alignment path is now evaluated for statistical significance (represented as *z*-score). This is done by evaluating the probability of finding an alignment path of the same length with the same or smaller number of gaps and distance from a random comparison of structures. Similarities with a *z*-score lower than 2 are spurious. The calculated *z*-score for 3qlb structure was 4.8 and that of all the other aligned protein structures was above 3. RMSD between BauA and FptA was calculated as 1.1. RMSD between BauA and other iron uptake proteins in Figure 4 was <2.

### 3.10. Data Validation

**Figure 5:**
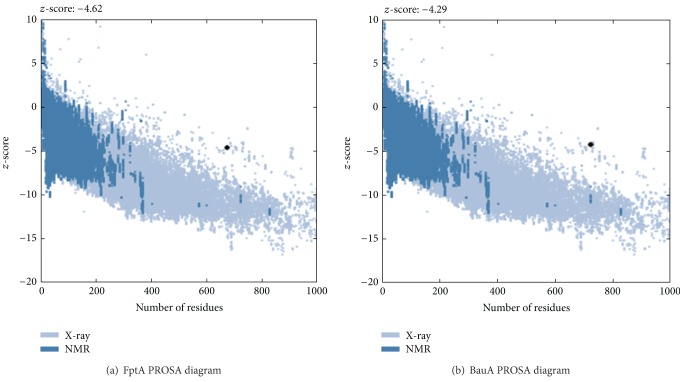
Comparison of data to validate the results on the BauA protein structure.

Figure 5 shows the data validation results. In depicted plots for BauA model and FptA structure the calculated *z*-scores are almost equal and within the range of scores typically found for native proteins of similar size. 

### 3.11. Ligand Binding Site Predictions

**Figure 6:**
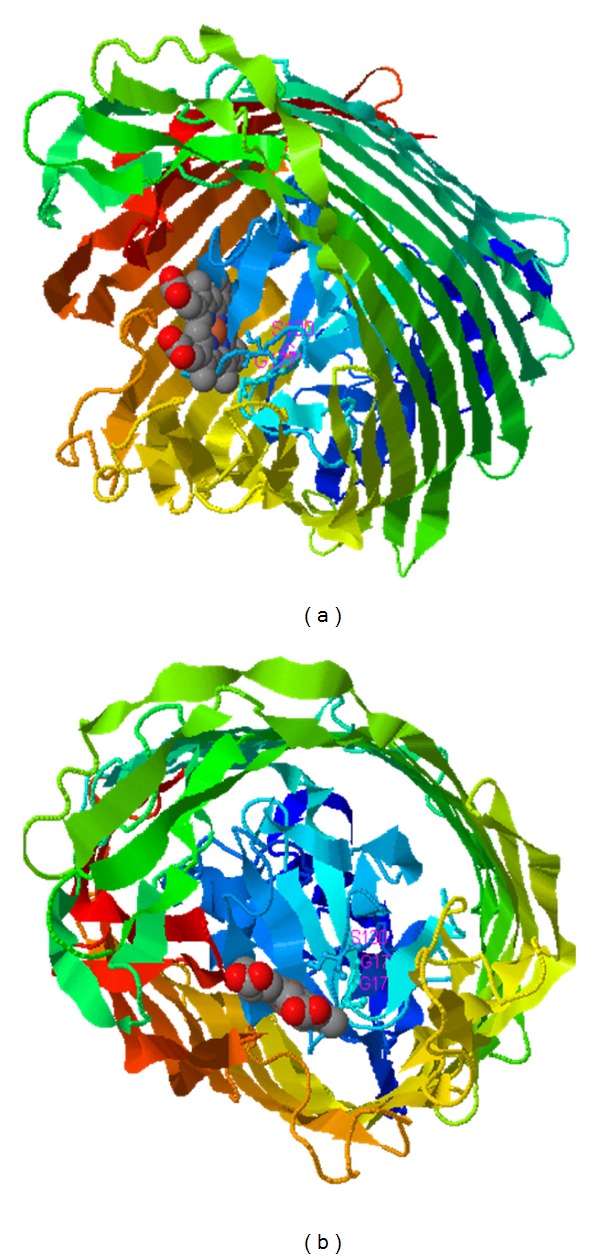
BauA ligand binding sites predicted by COFACTOR. (a) and (b) show BauA structure in contact with siderophore ligand from lateral and top views, respectively. BauA is shown in ribbon and ligand in the space filling model. Conserved residues especially R(67), W(68), and F(93) from cork domain and G(303), L(305), and D(344) from barrel are involved in iron binding site.

Ligand binding sites determined indicated involvement of conserved residues especially R (67), W (68), and F (93) from cork domain and G (303), L (305), and D (344) from barrel in iron binding site (Figure 6).

### 3.12. Cleft Analyses

Profunc suggested a region with average depth 22.45 Å, accessible vertices 77.34 Å, and buried vertices 18.05 Å, is the largest and deepest cleft in this protein.

## 4. Discussion

*A. baumannii* is one of the deadly bacteria in nosocomial infections [45]. BauA is the most important member of OMPs which uptakes acinetobactin, the siderophore of *A. baumannii*, in a complex with iron under iron limited conditions [6]. Functional blockade of the protein could have a cidal effect on the pathogen [6]. This study was conducted to develop 3D models of BauA by invoking various *in silico* methods such as homology modeling, threading, and combination of them as well as *ab initio*. Biochemical functions of proteins are generally acquired by their structures [46]. Linear chains of amino acids adopt a unique three-dimensional structure in their native environments [9]. Protein structures could be assessed by experimental and theoretical approaches. Bioinformatic approaches are noteworthy owing to snags before experimental determination of 3D protein structures [27, 46]. Our BLAST results showed that BauA exists in all pathogenic strains of *Acinetobacter* species. BauA antibodies cross-react with a range of *Acinetobacter* isolates for high similarity reason. Assessed crystal structures of several members of IROMPs family [3, 44, 47] provide important clues concerning the architecture of all TonB-dependent receptors including BauA, an important pathogenicity factor in *Acinetobacter baumannii *infections. In this regard, BauA sequence served as a query for BLAST search against protein data bank (PDB) to find the best template. In addition to *E* value, query coverage and max. identity are also involved in max. score definition. Lower *E* value and higher query coverage and max. identity are appropriate criteria for the selection. Thus, a hit with the highest total score could be the most reliable template. The use of some sequence alignment methods to identify a relationship between the target sequence and one or more possible templates is the first step in structure prediction [13]. Based on BLAST search and alignments generations, the predicted 3D model of the BauA could be applied to all BauA proteins in *Acinetobater*.

BauA has 142 residues conserved in TonB-dependent proteins of which 40 are located in cork domain. This phenomenon suggests that 20% of the domain is conserved. Other conserved residues were identified in barrel domain. In barrel 64 conserved residues were located at the beta-strands (21.55%), 35 in loops (16.91%), and 3 in turns (6.67%).

Therefore *β*-strand is the major matching region between the BauA and FptA in the secondary structure. As the model is predicted as a *β*-barrel, it is observed that the conserved residues were mostly in *β*-strands. The big gaps in the alignment were located in the outer membrane loop regions as these regions have a high degree of variation.

 Accuracy of prediction depends on the degree of sequence similarity. If a structure template with sequence identity of >50% is found for a query protein, homology modeling could be chosen as the best *in silico* method with an accuracy equal to low-resolution X-ray predictions [9]. When template and query sequences share 30%–50% identity, more than 80% of the C-atoms can be expected to be within 3.5 Å of their true positions. Significant errors would occur in prediction when the sequences share less than 30% sequence identity [9]. Since identity between the query and its template sequence was 20% (<30%) in our study, we assumed that threading could be more powerful than the homology modeling. However, homology modeling had been employed by other researchers for TbpA in *Neisseria meningitides *despite low identity between template and target proteins [48]. This discrepancy persuaded us to compare 3D structures predicted by homology as well as threading approaches. For homology approach, FptA was used as structural template, based on BLAST search against PDB. Accurate model could not be obtained through standard homology modelling techniques if low sequence identities exist between BauA and its template. Topology of IROMPs could be employed as a supplementary data for the purpose [48]. Twenty-seven transmembrane *β*-strands were detected when whole sequence served as query. The result could be validated by analysis on a related known protein as a control. Since structure of FptA, the most related protein, had been unveiled, its topology was predicted as a supplementary data. Only 22 transmembrane beta-strands were detected when the full-length template sequence was analyzed. The discrepancy could be due to performance criteria of the servers, because performance of results could be significantly decreased if full-length sequence is served [49]. The results would be more reliable by serving barrel sequence as an input data. On the other hand, BauA was classified as an IROMP based on homology analyses. Although the sequence identity between the proteins of the family is about 20%, all the receptors possess the same structural components; that is, transmembrane *β*-strands constructing barrel and about 200 amino acids in N terminus include cork domain [50]. Cork domain acts as a plug within the barrel occluding the opening of *β*-barrel. This domain is constituted from 4-5 antiparallel hydrophobic *β*-strands [50]. Error raised by applying full-length sequence of the protein could be due to hydrophobic propensity of amino acids laid at the strands. Start position of amino acids forming *β*-barrel is consensus as in the literature [50, 51]. The start position for BauA barrel is amino acid 192. When this fragment sequence served for topology prediction, the error was eliminated. Overall, it could be deduced that BauA is composed of a 22 antiparallel transmembrane beta-strands. Five more beta strands are constituting probable cork domain at the N terminal of the sequence. Prediction of the hydrophobic transmembrane regions in a protein sequence forming probable *β*-barrel could help determine the 3D protein structure [12, 24]. The topology confirms the selected template as well as predicted 3D structure.

Barrel of TonB-dependent receptors possess three main features: 10 short periplasmic turn ranging in length from 2 to 10 residues, the 22-stranded *β*-barrel, and 11 extracellular loops having 11 extracellular loops labeled from L1 to L11 for all of the transporters. The lengths of these extracellular loops can range from 2 to 37 residues consequently comprising roughly 40% to 50% of the total *β*-barrel [50]. These apply to BauA since it is a member of TonB-dependent receptors family. In this protein there exist 22 transmembrane *β* strands, 11 extracellular loops, and 10 periplasmic turns. *β* strands range from residues 7 to 21. The shortest *β*-strand is *β*2 with 2 conserved amino acids, and the largest is *β*7 with 3 conserved amino acids.

The lengths of extracellular loops range from residues 4 to 37. The shortest loop is L1 with 1 conserved amino acid, and the largest is L5 with 6 conserved amino acids. Turns range from residues 2 to 8. The shortest and the largest turns are T1 and T4, respectively. TonB spans the periplasmic space and physically interacts with siderophore receptors, resulting in energy transduction by a mechanism that is common to all TonB-dependent receptors [50]. In order to arrive at the most suitable model, the models built by various servers had to be evaluated. Scoring programs reflecting conformational energy were implemented in order to make a decision among predicted 3D structures. Since the *z*-score indicates overall model quality, its value must be compared with the *z*-scores of all experimentally determined protein chains in current PDB. It can be used to check whether the *z*-score of the predicted model is within the range of scores typically found for native proteins of similar size. Ramachandran plot, the second plot of the phi psi angles, is a standard tool exploited in determining protein structure [52]. LOMETS is a local threading metaserver, for protein tertiary structures predictions and spatial constraints [53]. Metaservers are paid more attention during recent years. They generate 3D structures by taking the consensus models from a variety of individual servers [34]. 3D structure, ligand binding sites, and cleft analyses denote that the cork domain forms a pair pocket within BauA barrel. The larger exterior pocket is open to the exterior environment and is restricted by cork domain, surface loops, and transmembrane strands. The borders of the smaller periplasmic pocket are cork domain loops and the barrel (Figure 6).

The Ramachandran plots and stereochemical/packing quality indices have been calculated by VADAR regarding the quality of the structures or viability of the folds. The stereochemical/packing quality index categorizes phi/psi and omega trends according the criteria given. These stereochemical quality indices allow specific “problem” residues to be rapidly identified. High-quality or high-resolution structure typically has scores close to 9 for all residues. Excluding volume is calculated using the Vornoi polyhedral method of Richards. Excluding volume represents the volume occupied by a residue as defined by its atomic radii and its nearest neighbors. Normally, if the protein is efficiently packed, all residues should have fractional volumes close to 1.0 ± 0.1. The excluded volume of 1 indicates acceptable values and good quality model.

One aim of ModRefiner is to draw the initial starting models closer to their native state, in terms of hydrogen bonds, backbone topology, and side-chain positioning [39]. The model refinement results in this study improved its quality (Figure 3). In the topology model presented here, external loop 5 is the largest loop and the side chains of all residues are highly exposed to the environment suggesting their role in initial binding events with Fe-siderophore complex. These regions could possess B-cell epitopes attractive for antibodies elicited against the protein. The RMSD measures the difference between C*α* atom positions between two proteins. The smaller the deviation is, the more spatially equivalent the two proteins are. Ideally, it should be 0.0 for two same proteins, but measurement errors and other variations cause deviation. Biological functions of proteins are performed in interaction with other molecules. Clefts size in protein surface could determine protein interaction with other molecules. Correlation of binding sites and clefts in a certain protein is a justification of our investigation on the clefts. A large cleft could increase opportunity for forming interactions between the protein and other molecules, particularly small ligands. Active site of a protein usually lies in the largest clefts or cavities. In over 83% of single-chain proteins, ligand is bound to the largest cleft. Thus, it is likely that protein ligand sites be identified by geometrical criteria alone [54]. Surface binding-pockets and occluded cavities are attractive for drug design and vaccine production against the pathogen. This important cleft located at exterior site of the protein structure includes 20 aromatic residues. This is the most aggregated location of aromatic residues. These features also appeared in the structure of the FptA-Pch-Fe receptor [44]. The model possesses characteristics relevant to the mechanism of iron translocation employed by BauA, although inferences made at this stage must be treated with caution. Access to the external surface of the plug domain is restricted to a cleft with an average depth of 22.45 Å. Although the model is a static representation of the receptor situated within a fluid membrane and the external loops are flexible, it is still hard to contemplate how a molecule as large as Fe-siderophore complex could be brought within the BauA binding pocket to allow iron-siderophore transfer. Although the *β*-barrel domain has a little sequence similarity with the outer membrane transporters BtuB, FecA, FepA, FhuA, FptA, and FpvA, their 22-stranded *β*-barrels are shown to be structurally similar when the C*α* backbones of the *β*-barrels of the outer membrane receptors are overlaid. The lengths of the extracellular loops vary between structures. The *β*-barrels are different in their lengths and widths which make their elliptical shapes vary.

The uptake of iron from transferrin, lactoferrin, hemoglobin, and siderophores has been identified in both Gram-negative and Gram-positive bacteria. The outer membrane in Gram-negative bacteria is a permeable barrier. Trimeric *β*-barrel proteins, porins, allow passive diffusion of small solutes with molecular weights less than 600 Da. [55] Ferric-siderophore complexes exceed porins molecular weight cutoff and thus specific outer membrane receptors are required for uptake into the periplasmic space. All of these iron uptake pathways involve an outer membrane receptor, a periplasmic binding protein (PBP), and an inner membrane ATP-binding cassette (ABC) transporter. The Gram-negative outer membrane lacks an established ion gradient or ATP to provide the energy for transport. This energy requirement is accomplished through the coupling of the proton motive force of the cytoplasmic membrane to the outer membrane via three proteins: TonB, ExbB, and ExbD [50]. OMPs are attractive as strong immunogens for vaccine design as well as diagnostic goals [56, 57]. Final structure of porins constructed from trimeric *β*-barrel could belong to quaternary structures of proteins. This issue suggests that tertiary structure of the proteins could be different from those of native structures. Since BauA is a monomeric protein, its tertiary structure could be more close to the native structure.

In conclusion, in contrast to the typical trimeric arrangement found in porins, BauA is monomeric. The barrel is formed by 22 antiparallel transmembrane *β*-strands (from *β*1 to *β*22). Loops connect adjacent strands; there are short periplasmic turns (from T1 to T10) and longer surface-located loops (from L1 to L11). All TonB-dependent receptors possess a short sequence of residues at the NH2-terminus termed as TonB box. It has been proposed that this region functions as a mediator of the physical interaction between TonB and TonB-dependent receptors. An N-terminal domain referred to either as the cork, the plug, or the hatch domain occludes the *β*-barrel. The structure of the cork domain of BauA like other TonB-dependent proteins such as FepA, FhuA, FecA, FpvA, FptA, and BtuB possesses a central mixed four-stranded *β*-sheet with surrounding loops and helices.

## Figures and Tables

**Table 1:** The first 10 hits with the highest scores of BLAST on the BauA sequence against protein data bank (PDB).

	Accession	Max score	Total score	Query coverage	*E* value	Max. ident.
1	3QLB_A	92.8	92.8	94%	3*e* − 19	24%
2	1XKW_A	82.0	82.0	83%	6*e* − 16	23%
3	2IAH_A	62.0	62.0	92%	9*e* − 10	22%
4	1XKH_A	59.3	59.3	83%	6*e* − 09	22%
5	1BY3_A	55.1	101	65%	1*e* − 07	25%
6	2FCP_A	53.1	99.7	65%	5*e* − 07	25%
7	1FI1_A	52.8	99.0	65%	7*e* − 07	25%
8	1QJQ_A	52.4	99.0	65%	8*e* − 07	25%
9	1FCP_A	52.0	92.4	72%	1*e* − 06	24%
10	1QFF_A	50.8	92.0	72%	3*e* − 06	24%

**Table 2:** Distribution of conserved amino acids in BauA.

Components	Total residues	Conserved residues	Conserved/ total residue
*β*1	9	1	11.11%
L1	4	1	25%
*β*2	7	2	28.57%
T1	8	0	0%
*β*3	11	4	36.36%
L2	11	1	9.09%
*β*4	11	2	18.18%
T2	5	0	0%
*β*5	11	1	9.09%
L3	33	5	15.15%
*β*6	15	1	6.66%
T3	4	1	25%
*β*7	21	3	14.28%
L4	19	2	10.5%
*β*8	17	3	17.64%
T4	2	2	100%
*β*9	17	2	11.76%
L5	37	6	16.21%
*β*10	15	1	6.66%
T5	3	0	0%
*β*11	17	5	29.41%
L6	11	0	0%
*β*12	12	3	25%
T6	3	0	0%
*β*13	11	2	18.18%
L7	20	2	10%
*β*14	12	5	41.66%
T7	5	0	0%
*β*15	11	5	45.45%
L8	22	2	9.09%
*β*16	14	5	35.71%
T8	3	0	0%
*β*17	16	6	37.5%
L9	19	2	10.5%
*β*18	8	3	37.5%
T9	7	0	0%
*β*19	15	1	6.66%
L10	10	5	50%
*β*20	13	5	38.46%
T10	5	0	0%
*β*21	8	1	12.5%
L11	21	9	42.85%
*β*22	10	3	30%

Total *β* strands	297	64	21.55%
Total loops	207	35	16.91%
Total turns	45	3	6.67%
Corck domain	197	40	20%

**Table 3:** Models built by various methods/servers and their evaluation results.

Method	Outlier	Allowed region	Favored region	Qmean	PROSA	Model/tool (template)
Template	1.4%	6.6%	92%	0.508	−4.62	3QLB
Homology modelling	1.3%	5.4%	93.3%	0.424	−4.04	ESyPred3D (3qlb)
Esy pred model by refine server	1.3%	4.6%	94.0%	0.429	−3.94	Esypred (3qlb)
Homology modelling	1.8%	7.0%	91.2%	0.401	−3.07	ESyPred3D
Homology modelling	2.4%	6.4%	91.3%	0.386	−3.9	(ps)^2^
Homology modelling	3.2%	7.6%	89.2%	0.398	−3.16	(Ps)^2^v^2^
Homology modelling	3.3%	7.3%	89.3%	0.359	−1.98	MODELLER (3qlb)
Homology modelling	9.9%	10.7%	79.4%	0.288	−0.24	3D-JIGSAW (3qlb)
Fold recognition	2.2%	4.0%	93.8%	0.448	−3.8	SPARKS^X^1
Fold recognition	2.4%	5.0%	92.7%	0.412	−3.03	SPARKS^X^2
Fold recognition	2.1%	5.4%	92.5%	0.437	−3.91	SPARKS^X^3
Fold recognition	2.6%	4.8%	92.5%	0.394	−3.97	SPARKS^X^4
Fold recognition	2.2%	4.8%	92.9%	0.417	−2.75	SPARKS^X^5
Fold recognition	2.5%	6.4%	91.1%	0.358	−1.75	SPARKS^X^6
Fold recognition	2.4%	5.0%	92.7%	0.399	−2.26	SPARKS^X^7
Fold recognition	3.0%	5.8%	91.1%	0.352	−1.79	SPARKS^X^8
Fold recognition	3.5%	7.9%	88.9%	0.402	−3.1	SPARKS^X^9
Fold recognition	3.3%	5.1%	91.6%	0.364	−1.78	SPARKS^X^10
Fold recognition and *ab initio *	4.3%	4.7%	90.9%	0.331	−1.8	Phyre2
Multiple-threading alignments	2.8%	5.4%	91.8%	0.426	−3.5	I-TASSER
Fold recognition	4.8%	7.4%	87.8%	0.329	−0.57	LOOPP1
Fold recognition	7.3%	8.8%	83.9%	0.343	0.33	LOOPP2
Fold recognition	5.3%	11.2%	83.5%	0.309	1.13	LOOPP3
Fold recognition	5.8%	8.5%	85.7%	0.289	0.92	LOOPP4
Fold recognition	6.4%	7.9%	85.8%	0.309	1.25	LOOPP5
Local meta-threading	1.5%	5.7%	92.8%	0.394	−3.6	LOMETS1
Local meta-threading	2.9%	5.4%	92.4%	0.374	−3.81	LOMETS2
Local meta-threading	2.1%	4.6%	93.4%	0.424	−3.81	LOMETS3
Local meta-threading	1.8%	5.0%	93.2%	0.419	−4.03	LOMETS4
Local meta-threading	2.1%	4.4%	93.5%	0.431	−3.87	LOMETS5
Local meta-threading	1.8%	5.1%	93.1%	0.417	−3.96	LOMETS6
Local meta-threading	1.1%	4.4%	94.5%	0.440	−4.02	LOMETS7
Local meta-threading	1.8%	5.1%	93.1%	0.417	−3.96	LOMETS8
Local meta-threading	1.8%	5.1%	93.1%	0.417	−3.96	LOMETS9
Local meta-threading	2.1%	5.0%	92.9%	0.401	−3.22	LOMETS10
Threading	1.8%	5.1%	93.1%	0.417	−3.96	MUSTER
